# On-Demand Gait-Synchronous Electrical Cueing in Parkinson's Disease Using Machine Learning and Edge Computing: A Pilot Study

**DOI:** 10.1109/OJEMB.2024.3390562

**Published:** 2024-04-18

**Authors:** Ardit Dvorani, Constantin Wiesener, Christina Salchow-Hömmen, Magdalena Jochner, Lotta Spieker, Matej Skrobot, Hanno Voigt, Andrea Kühn, Nikolaus Wenger, Thomas Schauer

**Affiliations:** Control Systems GroupTechnische Universität Berlin 26524 10587 Berlin Germany; SensorStim Neurotechnology GmbH 10587 Berlin Germany; SensorStim Neurotechnology GmbH 10587 Berlin Germany; Department for NeurologyCharité – Universitätsmedizin Berlin 14903 10117 Berlin Germany; Control Systems GroupTechnische Universität Berlin 26524 10587 Berlin Germany; Department for NeurologyCharité – Universitätsmedizin Berlin 14903 10117 Berlin Germany

**Keywords:** Edge computing, freezing of gait, inertial sensors, machine learning, on-demand cueing

## Abstract

*Goal:* Parkinson's disease (PD) can lead to gait impairment and Freezing of Gait (FoG). Recent advances in cueing technologies have enhanced mobility in PD patients. While sensor technology and machine learning offer real-time detection for on-demand cueing, existing systems are limited by the usage of smartphones between the sensor(s) and cueing device(s) for data processing. By avoiding this we aim at improving usability, robustness, and detection delay. *Methods:* We present a new technical solution, that runs detection and cueing algorithms directly on the sensing and cueing devices, bypassing the smartphone. This solution relies on edge computing on the devices' hardware. The wearable system consists of a single inertial sensor to control a stimulator and enables machine-learning-based FoG detection by classifying foot motion phases as either normal or FoG-affected. We demonstrate the system's functionality and safety during on-demand gait-synchronous electrical cueing in two patients, performing freezing of gait assessments. As references, motion phases and FoG episodes have been video-annotated. *Results:* The analysis confirms adequate gait phase and FoG detection performance. The mobility assistant detected foot motions with a rate above 94 % and classified them with an accuracy of 84 % into normal or FoG-affected. The FoG detection delay is mainly defined by the foot-motion duration, which is below the delay in existing sliding-window approaches. *Conclusions:* Direct computing on the sensor and cueing devices ensures robust detection of FoG-affected motions for on demand cueing synchronized with the gait. The proposed solution can be easily adopted to other sensor and cueing modalities.

## I. Introduction

Parkinson's disease (PD) is the second most prevalent neurodegenerative disease after Alzheimer's, with an estimate of over 61 million people worldwide [1]. Freezing of Gait (FoG) is one of the most disabling motor symptoms of PD, which emerges in the late stages of the disease, with a prevalence of 39.9 % [2]. It is characterized by episodic impairment or lack of motor capabilities despite the intention to walk [3]. PD patients describe FoG episodes “as if their feet were glued to the floor” [4]. The FoG episodes are of unpredictable and short nature, lasting only a few seconds, with prolonged episodes of longer than 30 seconds to be considered rare [5]. The FoG episodes can be categorized into subtypes based on their manifestation: akinesia defined as total lack of movement, festination referring to progressively smaller and frequent steps, shank trembling characterized by inability to move followed by tremor in the affected lower limb, and shuffling which is characterized by the inadequate lifting of the foot during a gait cycle [5]. Shuffling, festination, and shank trembling are the most prevalent subtypes, which often precede akinesia [6]. Due to its unpredictable occurrence and severity of FoG episodes, affected PD patients have a higher likelihood to fall, resulting in physical injuries, fractures, and death [7], [8].

In the last decades, external cueing has shown positive effects as a non-invasive treatment for motor symptoms in PD patients. Cueing refers to the presentation of external stimuli that serve as external targets or references for the execution of a movement [9]. Research suggests that cueing may help to reduce gait impairments and increase the mobility of patients, including improvements in step length, cadence, and gait velocity [10]. Furthermore, studies have noted a decrease in the occurrence of FoG [9], [11].

Various cueing modalities such as auditory, visual, somatosensory or combinations have been reported in the literature [12], [13]. It is worth noting that both auditory and visual cueing methods present certain limitations in real-life environments. Visual cues require subjects to concentrate on visual information, impacting their field of view and potentially straining their cognitive resources. Similarly, auditory cues may interfere with the natural sensory experience, affecting user-friendliness and comfort. Vibratory cueing may be distracting due to the audible sounds from vibration motors.

Electrical cueing is a compelling alternative. It stands out by not imposing any additional cognitive demands on subjects, as in the case of visual cues. Moreover, it avoids impeding the natural human sensory perception, a common concern with auditory cues. Furthermore, by adjusting the intensity of electrical cueing, a sensory or motor stimulation can be achieved. In the case of motor stimulation, a motor response is induced which can be beneficial for gait aspects, such as gait initiation and enhanced foot lift (e.g., foot drop stimulation).

Several studies reported on the positive effects of electrical cueing, indicated by improvements in gait performance and gait parameters, as well as in reducing FoG [14], [15], [16], [17], [18], [19]. In these studies, the prevailing form of cueing was gait-phase synchronous. The cueing was employed continuously without regard to the quality of gait mostly for limited time periods. However, for prolonged real-life application there are risks of habituation and dependence on the cues [20].

To address this issue, we introduce the first system for on-demand gait-synchronous electrical cueing. Our wearable system utilizes a single inertial sensor positioned mid-foot of the most affected leg to detect FoG episodes and triggers sensory electrical stimulation of the peroneal nerve during the swing phase of the gait. By detecting impaired movements and on-demand cueing, the system's aim is to enhance gait and prevent akinesia. The system builds up on our previous work on machine learning (ML)-based detection of FoG-affected foot motions by a single inertial sensor at one foot [21].

**Fig. 1.**
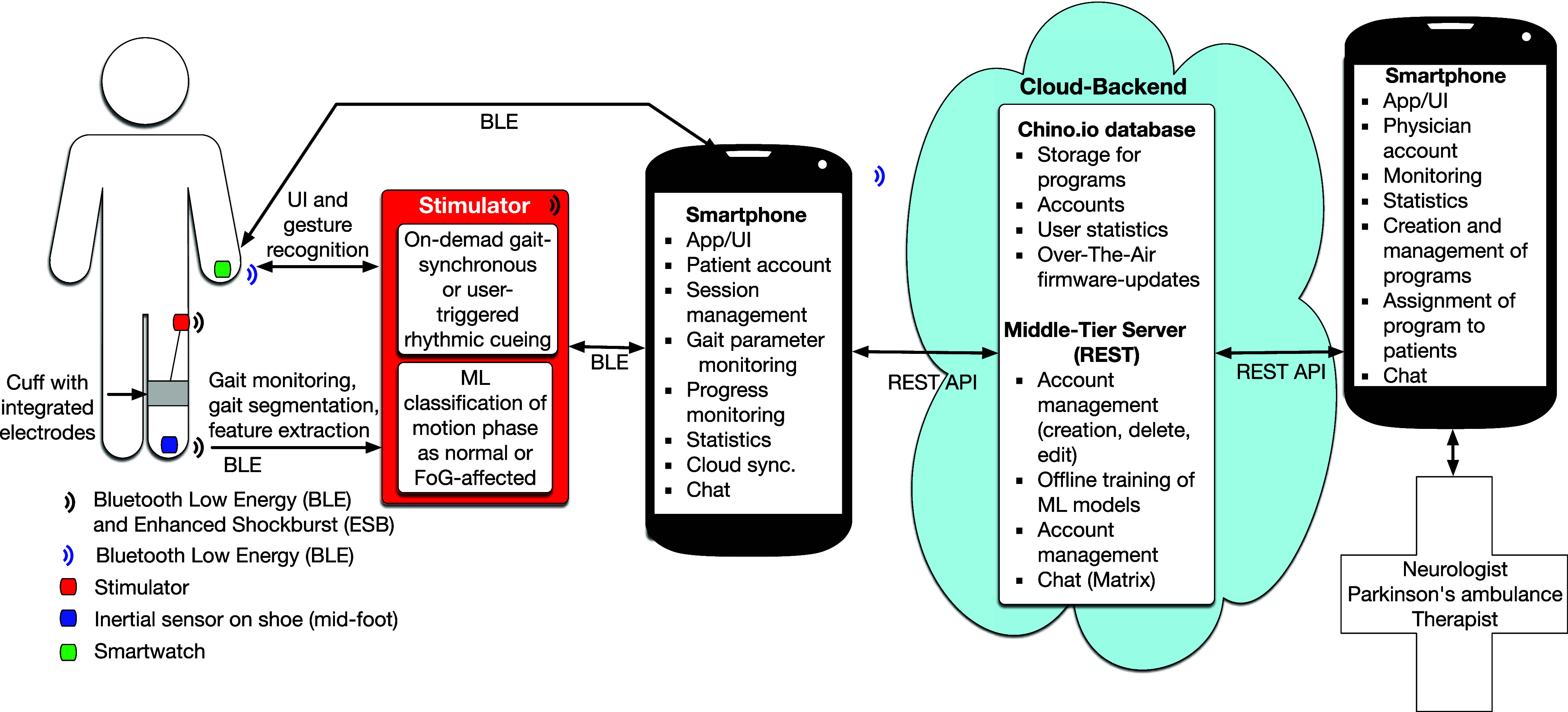
Overview of the system and its functionalities.

The most innovative aspect of our technical solution is the renunciation of the use of a smartphone for data processing between the sensor and the cueing device (stimulator). This leverages the usability and robustness in everyday life. To ensure adequate real-time performance and reliable operation, gait phase detection, feature extraction, classification and cueing control run distributed on the stimulator's and sensor's microcontroller units, wirelessly connected (edge-computing). Smart devices (watch and phone) with corresponding apps only serve as remote control to configure and supervise the system and offer cloud connectivity. This modern system architecture, illustrated in Fig. 1, is a unique feature of the gait assistant.

According to the reviews by Sweeney et al. [12] and Libero et al. [13], only Mikos et al. [22] have realized a similar standalone on-demand cueing devices without smartphone dependence for real-time operation. However, they did only evaluate with patients the FoG-detection performance of their sliding-window-based approach with large detection delay, but not the cueing functionality. In [22], the device with an inertial sensor and haptic cueing was placed at the shank, which makes foot movement detection more difficult. To demonstrate the functionality and safety of the system as well as the performance of gait phase and FoG detection, preliminary gait tests on two PD patients will be reported.

The following sections provide a comprehensive overview of the system, including its components, internal communications and functionalities. Two novel cueing concepts, involving on-demand gait-synchronous cueing and gesture-controlled rhythmic cueing, will be introduced. Subsequently, the gait test protocols and subsequent analysis of the gait phase and FoG detection will be outlined. In the results section, we report the technical performance and the observed changes in gait performance. Finally, the findings are discussed and conclusions are drawn.

## II. Materials and Methods

### A. System and Components

The system hardware consists of an inertial sensor (extendable to five), an electrical stimulator, a smartphone, and an optional smartwatch as well as a cuff with electrodes.

**Fig. 2.**
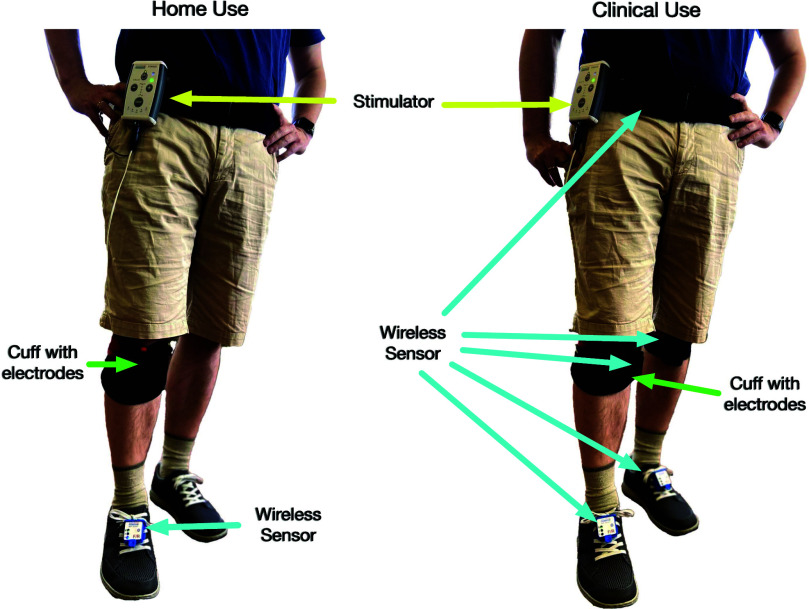
System with one foot sensor as mobility assistant for home use (left) and extended by four sensors (2 x lower leg, other foot, pelvis) for clinical environments (right).

The developed system offers two modes of configuration: as a home-use mobility assistant and as a clinical gait assistant and analysis system. In the first mode, a single sensor is required and attached to the foot of the most affected side of the body. For clinical use, up to five sensors can be utilized to provide additional gait analysis and therapy evaluation. The latter is out of the scope of this publication. Fig. 2 illustrates the setup for home use and clinical use.

We developed the inertial sensors based on a full inertial measurement unit (IMU) (BMX055, Bosch Sensortec GmbH, Reutlingen, Germany). The 12 b 3D acceleration and 16 b 3D rate of turn are sampled at 200 Hz. The acceleration range is set to $\pm$16 g and the range for angular velocities is set to $\pm 2000^{\circ }$/s. The core of the sensor device is the BMD-340 module (u-blox, Thalwil, Switzerland). It is a system-on-module (SoM) with integrated RF-transceiver and antenna that supports the Bluetooth Low Energy (BLE) 4.2/5.0 standard. Furthermore, the module's computing power is provided by an ARM Cortex M4 microcontroller unit (MCU) with 1 MB Flash, 256 kB RAM and a floating-point unit (FPU), allowing the implementation of complex algorithms on the sensor nodes. The device was complemented with a micro-SD unit to monitor and record the state of the patients over long periods of time. The sensor has compact dimensions of 35 mm × 35 mm × 15 mm and a lightweight design, weighing only 24 g. It is equipped with a 400 mA lithium-polymer battery.

The newly developed stimulator can generate charge-balanced rectangular biphasic pulses on up to 5 channels with a current amplitude of up to 100 mA. The pulse width $pw$ can be adjusted in the range of 30 $\mu$s to 1000 $\mu$s, and the frequency ranges from 1 Hz to 100 Hz, thus allowing both sensory and motor-peripheral stimulation. A multichannel stimulation is realized by sequentially connecting the output of the current source via a high-voltage switch matrix to the different electrode pairs. Similar to the sensor device, the stimulator uses the BMD-340 module for communication and processing in the application context. Additionally, a second dedicated 32 b ARM Cortex M4 microcontroller is employed for the time-critical generation and control of stimulation pulses and fault monitoring. The stimulator has a compact size of 120 mm × 80 mm × 30 mm, weights 185 g, and is equipped with a lithium-polymer battery (3200 mAh). An operating time of at least 6 hours is guaranteed for both the sensor(s) and the stimulator.

Two smart devices, an iOS Device (iPhone SE 2) and watchOS device (Apple Watch 5, 40 mm) were employed. We developed two applications, an iOS application as a primary user-interface for operating the system and a watchOS companion application.

Lastly, we designed a textile knee-cuff for integrating hydro-gel stimulation electrodes (UltraStim Snap Electrodes SN2020, 50 mm × 50 mm, Axelgaard Manufacturing Co., Ltd., California, United States) for a reliable positioning of the electrodes even by not experienced users. The cuff can accommodate two or three stimulation electrodes. The latter configuration allows for selective stimulation using two channels while sharing a common counter electrode.

### B. Communications

The communication between the system's components is realized via Bluetooth Low Energy (BLE), version 4.2. In the BLE connectivity, two roles can be distinguished, central and peripheral. Central devices are the ones that initiate the connection (scan for nearby devices and connect) and specify the parameters. Peripheral devices advertise their services and wait for connection request from the central device. The sensors assume the role of peripheral devices or servers. The smart devices act as central devices and clients. They initiate the connections with the stimulator and wait for data from the peripheral devices. The stimulator assumes both roles, peripheral and central. It acts as a central for the inertial sensors and as a peripheral from the perspective of the iOS and watchOS device. The sensors are only connected with the stimulator.

For the communication, the universal asynchronous receiver / transmitter (UART) protocol is used, on top of which a custom communication protocol was defined. A short connection interval of 7.5 ms was achieved between sensor(s) and stimulator. Events and data between sensor and stimulator can therefore be exchanged with an average latency of 7.5 ms. On the other hand, the connection interval between iOS/watchOS device and stimulator is constrained to 30 ms.

The communication between iOS and watchOS is achieved via the Apple Inc.’s proprietary communication interface Watch Connectivity. The communication between components of the system is event-based, meaning a packet will be sent if a specific event has occurred. Furthermore, stimulator and sensor(s) are time-synchronized with 1 ms accuracy using the parallel running proprietary communication protocol Enhanced-ShockBurst (ESB) developed by Nordic Semiconductor ASA (Trondheim, Norway).

As the communication with the backend has no time restriction, a REpresentational State Transfer (REST) protocol was employed. Fig. 1 shows the network topology and the technologies used for communication.

### C. Functionalities

The primary function of the proposed system is to provide an on-demand gait-synchronous sensory cueing with the purpose of aiding PD patients during their ambulation. To achieve a gait-synchronous cueing, a gait-phase-detection (GPD) algorithm, previously developed by the authors [21], has been implemented on the inertial sensor.

Three phases of Parkinsonian gait are detected regarding the foot: rest, unrest and motion. The motion phase (MP) is considered a sub-phase of the unrest phase. It indicates a displacement or orientation change of the foot. Assuming a healthy gait cycle, the motion phase corresponds to the swing phase, with toe-off and heel strike occurring at the beginning and end of the motion phase, respectively. Shuffling steps or heel lifting induced by shank trembling without forward movement are also counted as motion phases in the Parkinsonian gait cycle.

Feature extraction is implemented as another sub-functionality in the inertial sensor. Ten features (max. acceleration norm, max. pitch angle, min. pitch angle, stride length, max. stride velocity (in the transverse plane), turning angle, turned flag {0,1}, max. turn rate, mean turn rate, stride duration) are extracted at the end of each detected motion phase from one selected foot sensor (typically the foot of the most affected leg) and transmitted to the stimulator worn at a belt. Based on these features, the online classifier, implemented on the stimulator, assesses the motion phases as normal or FoG-affected. Depending on the classification result, on-demand cueing is automatically initiated. Characterizing the machine learning empowered system is its ability to process data in close proximity to its source, ensuring minimal processing delays – a defining feature of edge computing.

The global classifier underwent training on a dataset comprising 16 PD patients [21]. AdaBoost was used as the classification model, with decision trees as weak learners. The depth of the estimators was constrained to a maximal depth of three. Furthermore, ten estimators were trained. The dataset used for training and testing the model was collected at the Department of Neurology with Experimental Neurology, Charité – Universitätsmedizin Berlin and consists of 16 idiopathic PD patients (UPDRS-III 18 – 64, age 50 – 82 years). In a leave-one-subject-out cross-validation, the model showed a high performance of 87.5 % $\pm$ 8.2 % AUC (specificity of 78.8 %$\pm$11.9 %, sensitivity of 85.5 %$\pm$8.6 %, and accuracy of 84.0 %$\pm$7.0 %) [21].

**Fig. 3.**
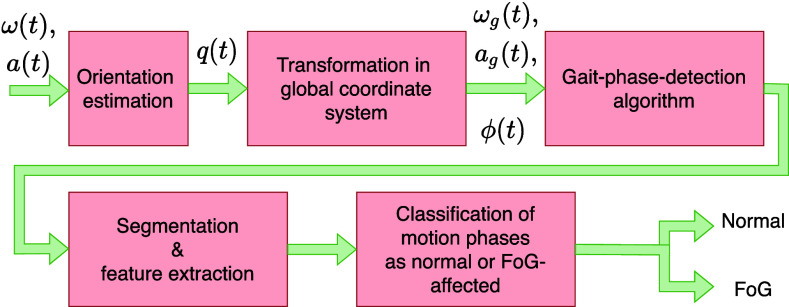
Overview of the different algorithms implemented on the system. $a(t)$ and $\omega (t)$ refer to the linear acceleration and angular velocities captured by the inertial sensor, respectively. The orientation $q(t)$, estimated as quaternion, is used to transform the input data to the global coordinate system ($a_\text{g}(t)$ and $\omega _\text{g}(t)$) and to extract the Euler angles $\phi (t)$.

Fig. 3 summarizes the different algorithms implemented in the system.

The primary functionality of the developed iOS application is to provide a user interface for both patients and physicians/therapists to control the system and cueing sessions, i.e. to select and start/stop a cueing program, and to control intensities. The companion watchOS application represents an easier alternative for keeping an eye on the system and enables gesture control of the system. The developed system differentiates between a patient and a physician/therapist user profile. Patients can run programs without changing stimulation parameters, except the stimulation intensities. On the other hand, physician/therapist accounts can create, edit and delete programs. In the context of digital health, physician/therapist accounts can assign programs to patients.

Monitoring and tracking the development of the PD is crucial so that the physicians/therapists can adjust the medication and or stimulation programs. In this context, physicians can track the daily gait performance of their patient by calling their detailed gait reports. Gait reports include metadata about the used stimulation program, intensities, and duration of usage as well as gait parameters such as number of steps, number of FoG-affected steps, percentage of FoG etc. The cloud backend of the system consists of two main components: the ISO 13485-certified Chino.io storage service for medical data and a middle-tier server. The backend utilizes the OAuth 2.0 standard for user account authentication, following the specifications outlined in RFC 6749. Chino.io is responsible for securely storing all programs, account data, patient data, and statistics. The middle-tier server handles various functionalities such as account management (creation and deletion), and role management.

### D. Cueing Concepts

It is reported that FoG is preceded by deterioration of gait parameters [6]. Therefore, on-demand cueing during motion phases impaired by FoG (i.e., in the presence of festination, shank trembling, and shuffling) might prevent the akinetic loss of mobility and restore normal motion phases by refocusing the attention on the gait. Should an akinetic episode nevertheless occur, standard rhythmic open-loop cueing (based on [17]) can be triggered by the patient on demand in the app or by an arm gesture with the watch to assist in exiting this state.

**Fig. 4.**
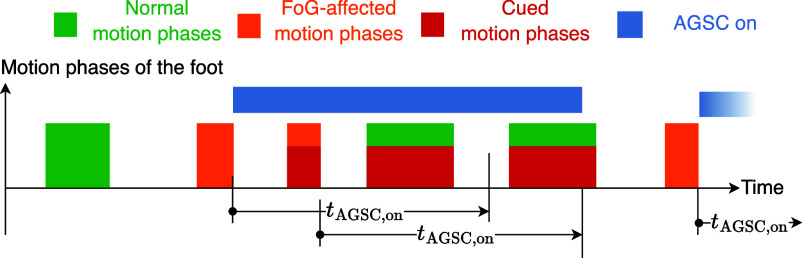
Example of an interval with adaptive gait-synchronous cueing based on motion phase classification.

On-demand cueing is delivered in case of a FoG-affected motion phase. If such a phase is detected by the classifier, a FoG episode is presumed for the next $t_\text{AGSC,on}$ seconds. During this on-demand cueing interval, every occurring motion phase will be cued (cf. Fig. 4). The interval is prolonged when a new motion phase inside the current interval is again identified as FoG-affected. The updated end time is then $t_\text{AGSC,on}$ seconds after the end of the latest detected FoG-affected motion phase. When the on-demand cueing is on, the cues, i.e. the sensory stimulation pulse trains, typically begin at the onset of the foot motion phases and terminate at the end of the motion phases. To avoid too short or too long stimulation pulse trains and too short pauses between pulse trains, the following additional restrictions apply:

• Between pulse trains must be at least $t_\text{off}$ seconds.
• The minimum duration must be $t_{\min}$ seconds.
• A pulse train must not be longer than $t_{\max}$ seconds.

The used parameters are listed in Table I.

**TABLE I:** Parameters for On-Demand Gait-Synchronous Cueing

Parameter	Values for the pilot study
$t_{\min}$	0.2 s
$t_{\max}$	0.8 s
$t_\text{off}$	0.2 s
$t_\text{AGSC,on}$	3 s
Stimulation frequency	50 Hz
Pulse width $pw$	300 $\mu$ s

(Adjustable Inside iOS App but Fixed for This Study)

**Fig. 5.**
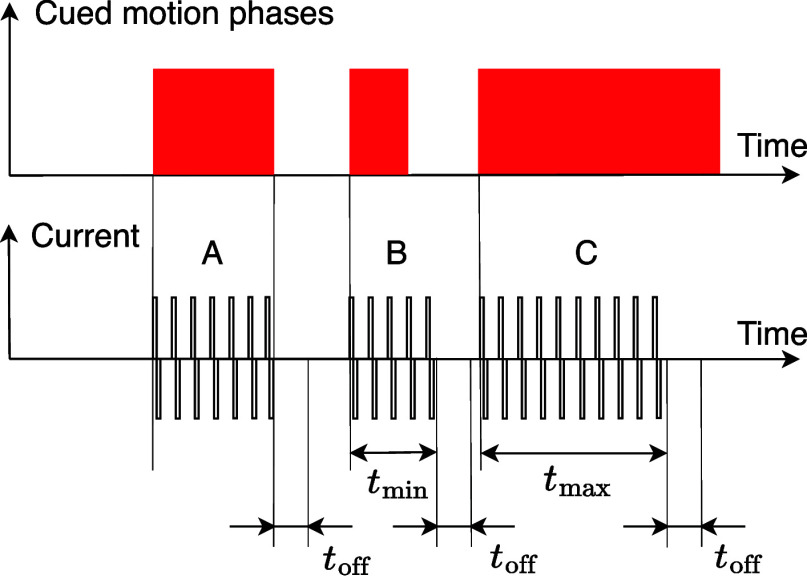
Pulse trains during enabled on-demand gait-synchronous cueing. A) MP duration is between $t_{\min}$ and $t_{\max}$, B) the MP lasts less than $t_{\min}$, and C) a MP longer than $t_{\max}$ occurs. During the $t_\text{off}$ periods, no new pulse train can be triggered by an MP. Individual current amplitudes can be set for both stimulation channels.

The minimum time was set after initial tests with healthy volunteers so that the cues could be readily perceived, while the maximum duration was based on observations of the maximum MP duration in patients with Parkinson's disease. Fig. 5

depicts the course of stimulation current during on-demand gait-synchronous cueing, assuming different durations of the motion phases.

### E. Tests With PD Patients

The feasibility, safety, and performance of the system were investigated with two female PD patients as part of an evaluation in accordance with the Medical Devices Act. The evaluation protocol received approval from the Ethics committee of the state office for health and social affairs Berlin (Ethikkommission des Landesamts für Gesundheit und Soziales (LAGeSo), reference: 21/0055-IV E 17, date: 16.06.2021) and was conducted by the Department of Neurology at the Charité–Universitätsmedizin Berlin, Germany. The patients had a confirmed diagnosis of idiopathic Parkinson's syndrome, as well as manifested gait disturbances and FoG symptoms. The patients used the system on one day, maintaining their usual medication.

A 10 m–walking test (10MWT) and the freezing assessment course (FAC) proposed in [23] were conducted. The 10MWT consists of walking a distance of ten meters, turning around, and returning to the starting point. The FAC consists of standing up from a sitting position, walking one meter to a marked square on the floor (40 cm × 40 cm) and turning 360$^{\circ }$ in both directions inside the square, afterward walking to a closed door, leaving the room after opening the door, returning to the chair, and sitting down. This test was designed to provoke FoG episodes, as scenarios conducted in narrow places (door) or obstacles can induce FoG.

Both tests were performed for each patient wearing the system under two conditions: without cueing (control condition; stimulation turned off) and with on-demand gait-synchronous cueing (stimulation condition). The order of the two conditions was different for the two subjects. Subject SC performed tests under the stimulation condition first and then under the control condition, whereas for subject CS it was the other way around. In the stimulation condition, sensory electrical cues were applied using one stimulation channel. The stimulation intensity, i.e. the current amplitude, was set for each patient individually, such that a sensory stimulation of the peroneal nerve was achieved, but no motor activities were induced (90% of the observed motor threshold). No further adjustment of the cueing parameters reported in Table I was conducted.

Under each condition (control or stimulation), the tests were performed three times. For the FAC, increased levels of additional motor and cognitive tasks were investigated in trial 2 and 3. During the second trial, the patient was asked to carry a tray while performing the task (dual-tasking). In addition to this, a cognitive task was added in the third trial, where patients were asked to count backwards in steps of seven.

The trials were recorded in one shot for each condition using two GoPro Hero (version 8 and 9) cameras (GoPro Inc. California, US) positioned at two different viewpoints ensuring continuous coverage of the subject's gait. For synchronizing the video and sensor data, a portable battery-powered device was used. This device could be manually triggered to deliver a time-aligned sequence of three vibrations and sounds. At the beginning of each trial, the device was pressed against a foot sensor and activated. The vibrations were captured by the inertial sensor, while the sounds were captured by the cameras.

To study the system's performance and the effect on the patient's gait, we captured for the trials the durations, the duration of cueing, the count of motion phases, the count of detected FoG-affected motion phases, as well as the number of stimulated motion phases. These parameters are assessed during the utilization of the system and are systematically recorded within the iOS app.

### F. Performance Analysis of Gait Phase and FoG Detection

To evaluate the detection of motion phases and the correct classification of FoG-affected and normal motion phases, a clinical expert annotated all motion phases of the foot, which was used to control the system in the synchronized videos (10MWT and FAC). In addition, FoG episodes, characterized by festination, shuffling, shank trembling, and akinesia were marked (not leg specific).

A labelled motion phase was defined to be detected correctly if one or more motion phases found by the system overlap, at least partially, exclusively with the annotated phase in the video. Otherwise, the labelled motion phase is not detected successfully. In addition, we counted incorrect motion phases found by the system that had no overlap with any labelled phase or that overlap with several labelled motion phases. For correctly detected motion phases, we determine the detecting delay for the start and end, as well as the duration difference between labelled and detected motion phase. If more than one found motion phase belongs to a labelled motion phase, only the first found motion phase is considered and the remaining neglected, as this first phase rules the pulse train generation.

For the on-demand cueing intervals, we further analyzed which temporal share of the motion phases was stimulated and which temporal share of the non-motion phases was stimulated. Here, the actual generation of the cueing pulse trains from the motion phases described above is considered.

To judge the motion phase classification, motion phases found by the system are automatically labelled as FoG-associated if their end time point is located in the expert-marked FoG episode; otherwise they are marked as normal. This ground truth has been compared with the outcome of the classifier to obtain the following patient-individual performance measures of the used pre-trained classifier [21]: sensitivity, specificity, and accuracy.

## III. Results

As the subject CS did not have a more severely affected leg, the patient was able to choose his preferred leg (the right) for gait phase detection and stimulation. For subject SC, the more affected right leg could not be used for stimulation due to a knee endoprosthesis and the left leg was selected instead. Both patients received the sensory-level electrical cues with 12 mA current amplitude. The observed gait parameters of the two subjects are reported in Table II.

**TABLE II:** Patient's Meta Data and Test Results for Both Conditions

	Patient CS		Patient SC	
Age	65		71	
Weight [kg]	50		81	
Height [cm]	158		160	
UPDRS-III	27		64	
L-Dopa [mg/d]	950		1522	
Condition	Control	Stimulation	Stimulation	Control
10-m Walking Test (all three trials)				
Duration (sum) [s]	98.7	77.1	116.4	120.5
Cueing (sum) [s]	–	23.7	43.9	–
# of MPs (sum)	98	81	109	119
# of FoG-MPs (sum)	20	10	32	40
# of cued MPs (sum)	–	21	41	–
Freezing Assessment Course (all three trials)				
Mean duration [s]	39.6	28.8	61.2	50.8
Duration [s] (sum)	118.9	86.5	183.7	152.0
Cueing [s] (sum)	–	49.4	155.9	–
# of MPs (sum)	115	88	192	152
# of FoG-MPs (sum)	69	39	142	91
# of cued MPs (sum)	–	58	170	–
Ziegler Score	13	6	30	24

Abbreviations: MP - Motion Phase, FoG-MP - FoG-Affected Motion Phase, # - Number

### A. Gait Phase Detection

For the left foot of subject CS, 301 foot-motion phases were marked in the videos, from which 283 (94.0 %) were correctly detected, 18 (6.0 %) were not detected (no overlapping detected motion phase was found), and 12 were falsely detected (no overlapping marked motion phases were found). For the left foot of subject SC, out of 170 motion phases, 162 (95.3 %) were correctly detected, 8 (4.7 %) were not detected, and 7 were falsely detected motion phases.

During all on-demand cueing periods for subject CS, the sensory stimulation covered 67.0 % of the duration linked to video-annotated motion phases, while also 7.5 % of the duration linked to non-motion phases have been associated with stimulation. For subject SC, the corresponding numbers were 63.1 % and 12.1 %, respectively.

**Fig. 6.**
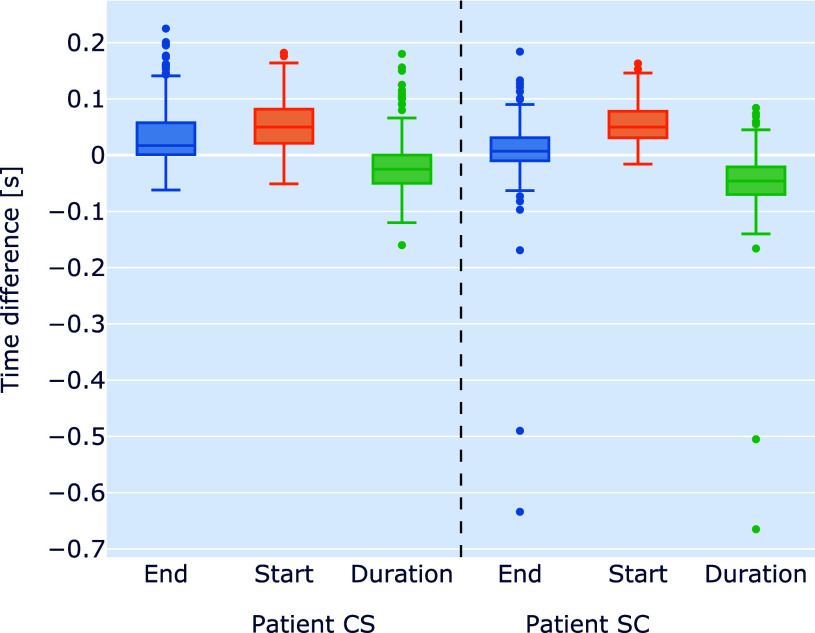
Box plot of delays of the start and end of detected motion phases compared with the annotated phases in the video data for both subjects. The differences in MP durations are also shown.

A delay analysis was conducted for validating the accuracy and reliability of our motion-phase detection system. By comparing the detected motion phases with the video-annotated motion phases, we gain insights into the temporal alignment between the automated detection and ground truth. Fig. 6 depicts the delay of start and end of the detected motion phases compared with the marked phases, as well as the difference in the duration for both subjects. According to Fig. 6, the start has the highest delay for both subjects. The median indicates a delay of approximately 0.05 s in the detection of motion start compared to the start of the motion phase marked in the video data. The median delay of the end of the motion phase is approximately 0.01 s. The median of the differences in duration between detected and marked phases is approximately −0.02 s and −0.05 s for subjects CS and SC, respectively.

The detected motion phases had a mean duration of 0.22 s and a standard deviation of 0.08 s over both subjects (minimal value of 0.015 s and maximal duration of 0.785 s).

### B. FoG Detection

Table III depicts the patient-individual performance of the system in classifying normal and FoG-affected motion phases. In summary, the system achieved results higher than 80 % in all three performance measures.

**TABLE III:** Performance Measures of the System in Detecting FoG-Affected Motion Phases (In %) for Stimulation (S) and Control (C)

Patient	Course	Trial	Sensitivity	Specificity	Accuracy
			S / C	S / C	S / C
SC	FAC	1	84.6 / 87.2	66.7 / 71.4	80.6 / 83.0
SC	FAC	2	88.9 / 70.6	66.7 / 82.4	81.5 / 74.5
SC	FAC	3	78.6 / 76.7	37.5 / 85.0	69.4 / 80.0
SC	10MWT	1	80.0 / 100	100 / 90.0	94.3 / 93.0
SC	10MWT	2	80.0 / 85.7	91.7 / 100	88.2 / 94.9
SC	10MWT	3	78.6 / 90.0	92.3 / 88.9	87.5 / 89.2
CS	FAC	1	91.7 / 100.0	93.3 / 70.0	92.6 / 84.2
CS	FAC	2	93.3 / 100	83.3 / 69.6	87.9 / 82.9
CS	FAC	3	77.8 / 94.1	84.2 / 80.0	82.1 / 86.5
CS	10MWT	1	60.0 / 71.4	88.9 / 92.6	84.4 / 85.4
CS	10MWT	2	50.0 / 50.0	100 / 96.2	92.3 / 92.9
CS	10MWT	3	50.0 / 40.0	95.5 / 91.6	91.6 / 82.8
Total performance based on					
summed confusion matrix			82.2	85.4	83.9

### C. Effects on Gait Tests

To investigate the effect of the proposed system on the patient's mobility, the gait parameters with and without stimulation must be compared. The on-demand cueing was only active in the stimulation condition. In this case, during the 10MWT and FAC, the cueing was active on average for 52.6 % (61.2 % for subject SC and 43.9 % for subject CS) with respect to the trial durations. Both subjects completed the 10MWT in a shorter time with less FOG-affected motion phases under on-demand gait-synchronous cueing. In the FAC trials, only subject CS showed a considerable reduction in completion time and Ziegler score, as well as in the required number of motion phases and their FoG proportion.

**Fig. 7.**
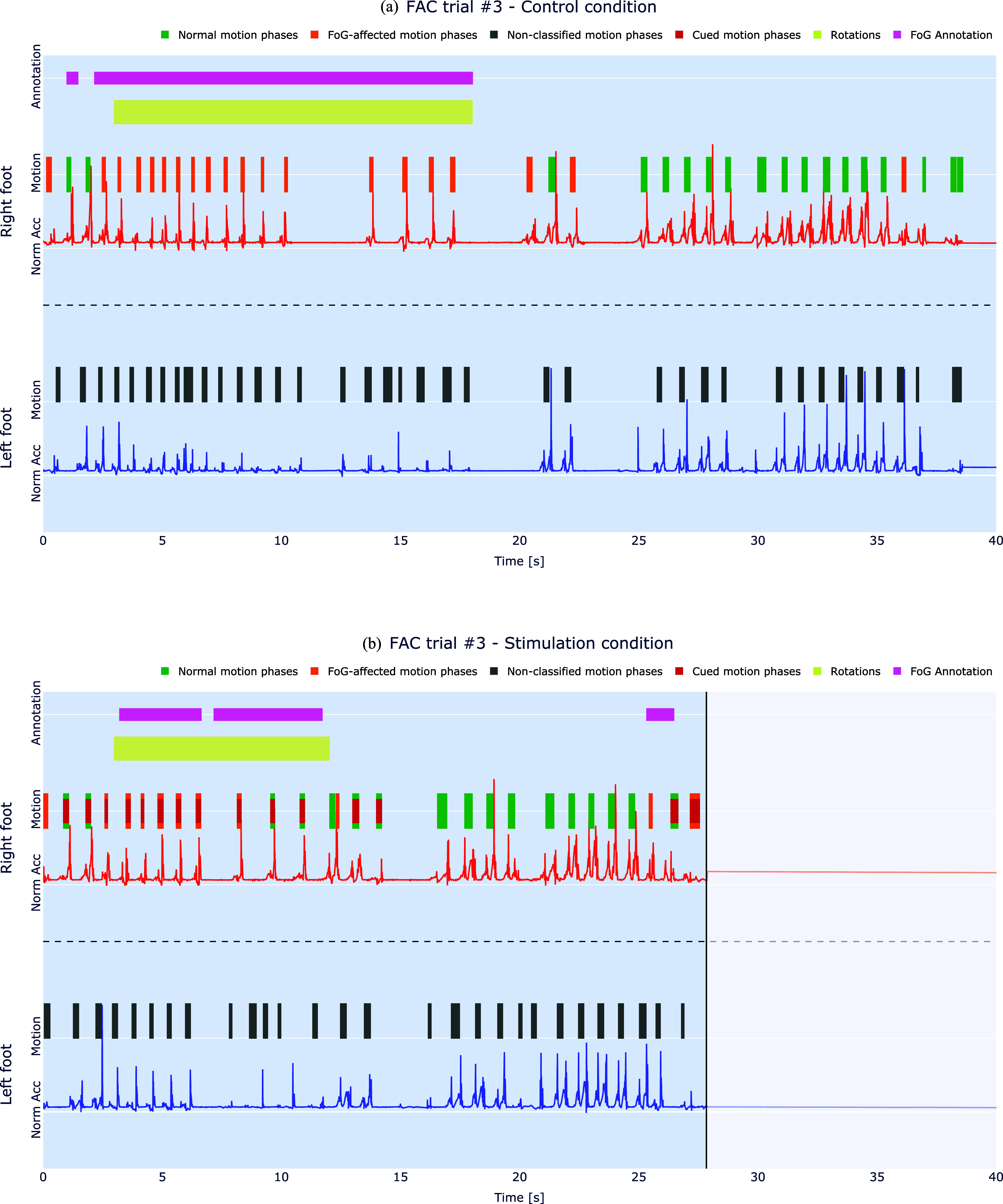
Functionalities of the developed system depicted exemplary for a FAC trial of subject CS under (a) control and (b) stimulation condition when carrying a tray and counting backwards. The rotations represent the turning tasks in both directions within the FAC test.

The functionality of the system is displayed exemplary in Fig. 7 for subject CS. Shown are FAC trials under control and stimulation condition when carrying a tray and counting backwards (trial 3). The figure shows the detected motion phases by the system for both legs (right leg used to control the stimulation, left leg assessed with the clinical sensor setup in which motion phases are stored on a SD card). The color used for the detected motion phases codes the category of the motion phase ($\color{green}{\text{green}}$: normal motion phases (MPs), $\color{Orange}{\text{orange}}$: FoG-affected MPs, $\color{red}{\text{red}}$: cued MPs, $\color{gray}{\text{gray}}$: non-classified MPs of the left leg). As explained in Section II-D, each FoG-affected MP starts an on-demand cueing interval with duration $t_\text{AGSC,on}$. During this interval, all detected MPs are cued, as shown by the MPs colored in red in Fig. 7, despite their classification. The completion time for the test under control condition was approximately 38.9 s and reduced under stimulation conditions to 28.3 s. The video-annotated FoG intervals of the clinical expert are shown in magenta for comparison. In addition, the periods, in which the patient is turning 360$^{\circ }$ in place, are indicated by the yellow bars. For the control condition, an akinesia episode is present between the two turns (at about 11 s).

## IV. Discussion

### A. Gait Phase Detection

The system possesses a high accuracy in detecting motion phases in individuals affected by PD. More than 94 % of the motion phases were correctly detected by the system. Most of the missed motion phases appeared during turns, when the foot is just turned without any heel-off. Detection of such motions would require precise yaw estimation for the foot orientation, which is not possible indoors due to drift in the estimated orientation and a disturbed reference (magnetic field).

The detection timing has been analyzed using the labelled videos. The delay in the detection of the end phase was low, indicating a fast response on heel strike. Contrary to this, the start detection has a higher time delay, resulting in a shorter duration of the detected motion phases than the marked ones.

### B. Gait-Synchronous Cueing

During on-demand cueing periods, the stimulation covered 63 to 67 % of the duration for the video-marked motion phases. The deviation from the desired 100% is mostly due to the delayed start detection. In addition, the stimulation also occurred in 7 to 12% of time intervals without motion phases. This is linked to prolonged cueing after the end of a motion phase due to a minimal required duration of the pulse trains of 0.2 s. As reported, many motion phases have been shorter than this set time period. Further research is needed to investigate the effects of choosing a shorter or longer minimal pulse train duration. A shorter duration might be problematic, as patients might not perceive too short pulse trains. The maximal allowed pulse train duration of 0.8 s has never been exploited, as the longest detected duration of a motion phase was 0.785 s. In conclusion, this safety parameter has been chosen correctly, retrospectively seen.

The pulse width and frequency of the pulse trains have been chosen similar to the ones used in foot drop stimulation. Further studies are required to investigate the best selection for those parameters concerning sensory cueing.

### C. FoG Detection

The reported classification performance corresponds well to our pre-work reported in [21] for the used pre-trained model, indicating a good generalization. In the evaluation of the classification performance in Fig. 7, it should be considered that the expert used both legs to mark FoG episodes, whereas the system relied on only one foot for FoG detection.

Huang et al. [24] presented a systematic review on FoG detection approaches. The performance of our developed system is comparable with the state-of-the-art approaches on unseen patients while using only a single sensor. For example, Milos et al. [22] achieved a performance of 86.2 % in sensitivity and 87.7 % in specificity using a sliding-window approach with delays in the seconds range. In contrast, our detection relies on gait segmentation, resulting in minimal detection latency determined by the gait cycle.

### D. On-Demand Cueing

It is evident in our data, that cueing is only applied if required (on-demand) and not permanently. During longer phases of straight walking, fewer cues have been administrated as less FoG is observed. During turns and door passages, as present most of the time during the FAC, cueing is more often activated in the two evaluated patients. The cueing effects observed in the two subjects were heterogeneous, and a larger subject population is necessary to draw final conclusions about the performance of the system. The subject CS, who showed improvements in both tests (10MWT and FAC), had a lower UPDRS-III score, a lower L-Dopa dosage per day, and could use the system on her preferred leg in comparison to the subject SC who did not benefit as much from the cueing. Apart from these possible reasons for the performance differences, also the order of the investigated conditions might have influenced the results. The system detects FoG only for one selected leg, and applies the electrical cues also only at this leg during motion phases. As shown in Fig. 7 for subject CS, the cues have a positive effect on the motion phases of the selected leg and the entire gait (faster walking and turning with fewer steps). However, one possible limitation may be that FoG-affected motion phases might still remain in the non-stimulated leg (also visible in the FoG episodes marked by the clinical expert). It remains unclear if swapping the stimulated leg or using bilateral cueing would have improved the gait performance even more for this patient. The current hardware and software architecture of our system allows a straightforward extension, so that bilateral FoG detection and cueing can be achieved. However, a second sensor would be required for capturing the movement of the other foot, slightly increasing complexity to the system.

### E. Usability, Latency, and Robustness

No direct performance metrics for the latency, usability, and robustness of the system have been used on the patients' data. However, it is a fact, that the smartphone is just a remote control to start and stop the system. Once started, the user can walk away and leave the smartphone behind. This is clearly an improvement in usability, and the risks of communication failures will be clearly reduced by avoiding unnecessary system communication. In terms of latency, we have performed internal measurements at the embedded system level and demonstrated that the average latency of events arriving at the stimulator from the foot sensor is only 7.5 ms. Between the smart devices and the stimulator or sensor, we could not establish a Bluetooth Low Energy connection interval below 30 ms. If the smartphone would be the central processing unit, an event from the sensor would require at least 60 ms to trigger stimulation. Concluding, our implementation approach reduced this average latency by 87.5%.

### F. Limitations

A limitation of the presented approach is that the detection of the FoG episodes is step-based, meaning that a step has to be made in order for the classification algorithm to be triggered. This has the drawback that akinetic episodes cannot be detected. In order to mitigate this drawback, classical rhythmic cueing is foreseen. It can be on-demand triggered by the user to help leaving the akinetic state. In the reported tests, this function was not needed and therefore not tested.

Enabling motor-level stimulation could render the proposed system to an on-demand foot drop stimulator. Such a change would provide additional foot lift to the patients that can improve the safety and performance of the gait additionally [19]. However, the current GPD should be extended/modified to enable earlier detection of heel-off, commonly used to trigger foot drop stimulation.

Electrical cueing also has limitations. Essential to its functionality is the necessity for consistent contact between the electrodes and the skin. Stimulation effects vary depending on the skin conductivity and external factors such as sweating.

The system should be ready for home use. However, additional usability studies could reveal problems that should be improved. This could be the handling of the foot-sensor attachment to the shoelaces by the patient, or the application of the cuff with the stimulation electrodes under long pairs of pants. So far, only shorts have been worn in the pilot study. Future improvements to the system for home use should include automatic detection of gait activity and human posture, e.g. to avoid unwanted stimulation when sitting. Integrated inertial sensors in the stimulator and in the smartwatch could be required and used for this purpose.

## V. Conclusion

A wearable system empowered by edge computing and machine learning has been developed, which can detect in real-time abnormal, FoG-affected motion phases of one foot directly on the sensing and cueing devices without relying on a smartphone. Two cueing concepts have been introduced: an on-demand gait-synchronous cueing to improve FoG-affected gait and prevent akinesia, and an arm-gesture initiated rhythmic triggered cueing to overcome remaining, unavoidable phases of akinesia. The system's functionality and safety have been investigated in a pilot study with two PD patients. The first results show good performances for foot motion detection (above 94% detection rate) and classification of foot motions as FoG-affected or normal (accuracy of 84%) as well as a tendency to reduce FoG, but larger randomized studies are necessary to confirm these findings. As demonstrated, cueing is only active when the gait pattern deteriorates. It is expected that this form of cueing prevents habituation effects and cueing dependence, problems known from open-loop continuous cueing. Therefore, the system is potentially suitable for the future as an assistive technical aid at home, requiring solely a single sensor at one foot, a shank cuff and a stimulator (similar to existing and well-accepted foot drop stimulators). The usage of smart devices and apps as remote control offers the integration of telemedicine functionality to monitor the gait performance and adjust stimulation parameters.

## Author Contributions

AD contributed to the development of on-demand cueing system, with a primary focus on software and algorithms development for edge devices and iOS/watchOS applications. Additionally, AD contributed to the conceptualization and implementation of wireless communication using BLE in edge devices and to both the conceptualization and development of the backend. The hardware was made available to the authors by SensorStim Neurotechnology GmbH (CW, HV). TS advised on the developing process. MJ, CSH, LS, MS, NW conducted the tests with Parkinson's patients in clinic, as well as data recording and annotating. AK, TS and NW: funding acquisition and supervision. All authors contributed to the article and approved the submitted version.

## Conflict of Interest

AD is employee and TS, CW, HV are co-founders of the SensorStim Neurotechnology GmbH, which is a company developing sensor-based stimulation devices. All other authors declare that they have no competing interests.
